# Emerging preclinical pharmacological targets for Parkinson's disease

**DOI:** 10.18632/oncotarget.8104

**Published:** 2016-03-15

**Authors:** Sandeep Vasant More, Dong-Kug Choi

**Affiliations:** ^1^ Department of Biotechnology, College of Biomedical and Health Science, Konkuk University, Chungju, South Korea

**Keywords:** dopaminergic, Parkinson's disease, pharmacological targets, neuroprotection, preclinical

## Abstract

Parkinson's disease (PD) is a progressive neurological condition caused by the degeneration of dopaminergic neurons in the basal ganglia. It is the most prevalent form of Parkinsonism, categorized by cardinal features such as bradykinesia, rigidity, tremors, and postural instability. Due to the multicentric pathology of PD involving inflammation, oxidative stress, excitotoxicity, apoptosis, and protein aggregation, it has become difficult to pin-point a single therapeutic target and evaluate its potential application. Currently available drugs for treating PD provide only symptomatic relief and do not decrease or avert disease progression resulting in poor patient satisfaction and compliance. Significant amount of understanding concerning the pathophysiology of PD has offered a range of potential targets for PD. Several emerging targets including AAV-hAADC gene therapy, phosphodiesterase-4, potassium channels, myeloperoxidase, acetylcholinesterase, MAO-B, dopamine, A_2_A, mGlu5, and 5-HT-_1A/1B_ receptors are in different stages of clinical development. Additionally, alternative interventions such as deep brain stimulation, thalamotomy, transcranial magnetic stimulation, and gamma knife surgery, are also being developed for patients with advanced PD. As much as these therapeutic targets hold potential to delay the onset and reverse the disease, more targets and alternative interventions need to be examined in different stages of PD. In this review, we discuss various emerging preclinical pharmacological targets that may serve as a new promising neuroprotective strategy that could actually help alleviate PD and its symptoms.

## INTRODUCTION

Parkinson's disease (PD) is the most common neurodegenerative disorder after Alzheimer's disease (AD), affecting > 1.5% of the global population. PD is characterized by progressive loss of dopaminergic neurons in the substantia nigra pars compacta (SNpc) region of the brain. The incidence of PD increases with age, and mean age of onset is about 65 years. Fresh evidences are insinuating towards epigenetic changes such as histone modifications as one of the leading factors behind brain aging and PD [1]. The lack of dopamine (DA) causes the classical motor symptoms of bradykinesia, rigidity, and resting tremors [2]. However, other neuronal fields and neurotransmitter systems including the locus coeruleus, the dorsal motor nucleus, the substantia innominata, the autonomic nervous system, and the cerebral cortex are also involved in PD [3]. As a consequence of this other symptoms including cognitive decline, sleep abnormalities, depression, and gastrointestinal and genitourinary disturbances are also developed. These “non-motor” symptoms progress and dominate in advanced stages of PD. It is projected that the number of patients with PD will increase to 8.7 million by 2030. Men are more susceptible to PD than women [2]. Years of genetic investigations in PD have led to the discovery of several heritable and sporadic forms of this disorder. The typical form of PD occurs in a sporadic idiopathic fashion [4] and hereditary factors are minimally associated with early-onset PD [5]. Comprehensive analyses of patients with mutations in *PINK1*, *DJ1, LRRK2, UCHL1, MAPT, GBA, NAT2, INOS2A, GAK, HLA-DRA, APOE, and SNCA* have significantly increased our understanding regarding the morphological, and pathological aspects seen in clinical and preclinical stages of PD [6, 7]. Other anticipated causes include environmental toxins, medications, and viruses that result in increased oxidative stress [8, 9]. Patients are classically diagnosed with PD based on thorough neurological and physical examinations. The diagnosis is made when two of the four classical symptoms of PD are present [10]. An accurate PD diagnosis is aided by DA transporter single photon emission computed tomography [11]. However, sensitivity and accuracy of this scan for diagnosing PD are equal to a clinical diagnosis; thus, raising doubts about the benefits of using a brain scan to confirm the diagnosis [12]. Despite these advances, a post-mortem neuropathological examination of brain tissue is the ultimate choice to precisely confirm the diagnosis of PD [13].

Levodopa (L-DOPA) was introduced almost 40 years ago and remains the best functional therapy for decreasing PD symptoms. However, the effects of L-DOPA tend to decrease as the disease advances. It gradually becomes difficult to manage symptoms and patients invariably develop motor impediments that comprise of motor fluctuations, dyskinesia, freezing, and fall [14]. Numerous other drugs from different classes are available to treat PD, and usually involve layering different treatments in a polypharmaceutical approach. More advanced DA agonists, such as pramipexole and ropinirole approved in 1997, were designed to selectively stimulate DA 2 receptors. Other key drug classes including the catechol-O-methyltransferase (COMT) inhibitors and monoamine oxidase (MAO) inhibitors have been approved to treat PD. As such, no single treatment is considered completely efficacious throughout the progression of PD; therefore the treatment is addressed according to the patient's disease severity and progression [15]. However, due to the side-effects associated with L-DOPA, nearly half of PD market sales are driven by advanced DA agonists along with COMT and MAO inhibitors. Nevertheless, the availability of generic versions of the DA agonists' pramipexole and ropinirole will limit growth across all major markets. Competition from generic compounds is most prominent in the United States, and it will remain the biggest market for PD until 2019. Germany is projected to produce the highest sales for PD drugs in 2019, whereas France will remain the smallest PD market until 2019 [15]. Symptomatic therapies will not take the place of existing drugs even though the market will rise due to new therapies in the pipeline. However, the existing landscape of therapeutic options will be revolutionized with development of neuroprotective candidates. Disease-modifying therapies for PD will change once the target genes and proteins that mediate the degeneration of dopaminergic neurons in the SN are identified. Nevertheless, it will take another decade until significant disease-modifying targets are identified [15]. Several emerging targets including AAV-hAADC gene therapy, phosphodiesterase-4, potassium channels, myeloperoxidase, acetylcholinesterase, MAO-B, DA, A_2_A, mGlu5, and 5-HT-_1A/1B_ receptors are in different stages of clinical development. In this review, we discuss various emerging preclinical pharmacological targets that may serve as a new promising neuroprotective strategy that could actually help alleviate PD and its symptoms.

## IMPORTANCE OF TARGET IDENTIFICATION AND VALIDATION FOR BETTER DRUG DISCOVERY

Discovering a new target from an original idea and launching the final product is a multifaceted process that involves around $1 billion and can take 12-15 years. Awareness of a target can come from various sources including academic, clinical, and commercial sectors. Once the target is selected, the therapeutic industry and academic centers develop a number of early procedures to identify lead molecules that possess appropriate characteristics for acceptable drugs [16]. Two main reasons contribute to drug failure. The first is a lack of safety and the second is ineffectiveness in humans. Therefore, a key step for developing a new drug is identifying and validating the target. A target is a comprehensive term applied to a variety of biological entities, including enzymes, substrates, metabolites, receptors, ion channels, transport proteins, DNA, RNA, ribosome, monoclonal antibodies, and various physiological mechanisms [17]. A target must be safe, potent, and efficacious to be functional; it should also satisfy clinical and commercial needs and should be druggable. A “druggable” target means it is available for developing a putative drug molecule. The molecule can be a small or large biological molecule but should elicit a biological response that can be measured *in vitro* and *in vivo*. Certain target classes are more amenable to discovering small molecule drugs, such as G-protein-coupled receptors, whereas antibodies are better at blocking protein/protein interactions. Proper identification and validation of the target allows us to learn the role of target modulation during mechanism-based side effects. Bioinformatics is one of the prime technique that helps to identify, choose, and prioritize probable disease targets [18]. The literature has a variety of sources, including publications, patent information, proteomics data, gene expression data, transgenic phenotyping and compound profiling data. Additional approaches for identification include investigating mRNA/proteins and their role under pathological conditions. Another excellent method is to look for genetic polymorphism and its consequence on disease progression. [19]. Furthermore, mutations in human phenotypes can nullify or exacerbate certain receptor, such as mutations in voltage-gated sodium channel NaV1.7; results in insensitivity or oversensitivity to pain [20, 21]. Phenotypic screening is another method that can be used to recognize pertinent disease targets [22].

Validation techniques for drug targets include using *in vitro* and *in vivo* tools and modulating a preferred target under disease conditions [23]. Reliability of these results increases significantly using a multi-validation approach [16]. Antisense technology is a powerful method that uses chemically modified RNA-like oligonucleotides designed to be complimentary to a region of a desired mRNA molecule [24]. The effects of antisense oligonucleotides are reversible compared with the gene knockout approach, and sustained presence of the antisense is required to inhibit the desired protein [25]. Nevertheless, the chemistry associated with synthesizing oligonucleotides has resulted in molecules with restricted bioavailability and strong toxicity, making *in vivo* use problematic [16]. As an alternative, transgenic animal are an attractive option for validation, as phenotypic changes resulting from gene manipulation can be observed. However, using transgenic animals is time-consuming and expensive. Hence, small interfering RNA (siRNA) has become popular for target validation. However, delivery to the target cell can be a problem with siRNA methodology [26]. Monoclonal antibodies are an another brilliant target validation tool, as they bind with a major region of the desired molecule surface, and thus help to selectively bind the target with high affinity. The only disadvantage of this technique is that antibodies cannot cross the cell membrane and cannot bind intracellular targets. Recently, chemical genomics has emerged as a systemic application of molecules to target identification and validation. Chemical genomics is nothing but the study of genomic responses to chemical compounds. This approach helps early identification of novel targets and drug [27].

## EMERGING NEW PRECLINICAL EVIDENCE OF TENTATIVE PD TARGETS

Existing PD therapy include re-formulating conventional drugs already approved for PD, chemically modifying compounds that are accepted for other indications, developing novel small-molecules and gene therapy-based approaches. The pipeline for the therapeutic development of new targets appears to be dynamic on the surface. However, if conventional dopaminergic therapy is removed from the picture, the existing landscape is far less promising [28]. Most of the drugs approved for PD do not have adequate efficacy nor do they avert disease progression. Consequently, there is a critical need to discover novel therapies to overcome the disadvantages associated with current therapies. In the following section, we will discuss some emerging pharmacological PD targets with substantial preclinical evidence (as also depicted in Table 1) that can be further considered for clinical investigations to develop new PD therapeutic.

**Table 1:** Emerging preclinical pharmacological targets for Parkinson's disease (PD)

Target	Pharmacological Model	Experimental Outcomes	Ref.
BAG1	SH-SY5Y cells and C57BL/6J mice	BAG1 protects against mutant α-syn and rotenone-induced cell death. BAG1 also protects dopaminergic cells in the SN against MPTP-induced toxicity *in vivo*.	[279, 280]
Adenosine A2A Receptor	MPTP-intoxicated Marmosets	Istradefylline (Adenosine A2A Receptor antagonist) effectively alleviates motor impairments in combination with low dose dopaminergic drug without aggravating dyskinesia.	[281, 282]
5Alpha-Reductase	MPTP-intoxicated Mice	Dutasteride (5Alpha-Reductase inhibitor) significantly prevents the demise of dopaminergic neurons in MPTP-intoxicated mice.	[283, 284]
CB2 Receptor	Intrastriatal injections of LPS into C57BL/6J, male wild-type or CB2 knockout C57BL/6J mice	Genetic deletion of CB2 receptors exacerbates LPS-induced inflammation. Stimulating CB2 receptors with HU-308 decreases the LPS-induced proinflammatory response.	[285, 286]
Cyclin D3/CDK6	6-OHDA-induced toxicity in SH-SY5Y cells	Sodium butyrate and suberoylanilide hydroxamic acid stabilizes the proliferative activity of PD lymphoblasts and decreases 6-OHDA-induced cell death in neuronal cells by preventing over-activation of the cyclin D3/CDK6/pRb cascade.	[287]
PDE7	Ex vivo cultures obtained from male Wistar rats	Inhibiting PDE7 induces proliferation and growth of embryonic ventral mesencephalic-derived neurospheres and adult progenitor cells *in vivo* in the SNpc, as well as increases Nurr1 and TH MAP-2 expression in neural stem cells obtained from ventral mesencephalon.	[288, 289]
SIRT2	LUHMES cells and MPTP model of PD	AK7 (SIRT2 inhibitor) protects dopaminergic neurons against α-syn-induced neurotoxicity in differentiated LUHMES cells and in MPTP model of PD. AK7 prevents dopamine loss, encourages long-term endurance of dopaminergic neurons, and conserves functional performance.	[290, 291]
Trib3	PC12 cells and rat dopaminergic ventral midbrain neurons exposed with 6-OHDA, MPP^+^, or α-syn fibrils	Toxin-induced upregulation of Trib3 protein increases neuronal cell death. Trib3 knockdown protects PC12 cells and ventral midbrain dopaminergic from all toxins.	[292, 293]
SK channel	Rotenone intoxication of human post mitotic dopaminergic neurons	Stimulating SK channels decreases mitochondrial membrane potential and maintains cell viability, the dendritic network, and ATP levels after rotenone insult.	[294, 295]
Sigma-1 Receptors	Intrastriatal injection of 6-OHDA in mice	PRE-084 (sigma-1 receptor agonist) facilitates steady and substantial development of impulsive forelimb use and augmented density of dopaminergic fibers in the utmost denervated striatal regions.	[296, 297]
Ribosomal protein s15	*Drosophila* and human neuronal PD models	Phosphorylation of ribosomal protein s15, a substrate of LRRK2, is essential for the toxicity-related effects of the common G2019S LRRK2 mutation in human dopamine neurons and in G2019S the LRRK2 mutated *Drosophila* model of PD.	[298, 299]
Prolyl Hydroxylase Domain	Inducible genetic dopaminergic glutathione depletion model	Antagonizing the prolyl hydroxylase domain pharmacologically via 3,4-dihydroxybenzoate substantially lessens mitochondrial dysfunction and damage to dopaminergic neurons in the SNpc.	[300, 301]
VDR and NMDAR	Haloperidol-induced Parkinsonism in mice	Vitamin D3 treatment enhances neural activity, motor-cognitive function, glia/neuron survival, and expression of neurofilaments. Inhibiting NMDAR and co-treatment enhances motor-cognitive functions but not as much as values detected post VDR stimulation.	[302, 303]
LHb	6-OHDA-induced PD model	LHb lesions decrease apomorphine-induced rotational behavior. The lesions also increase dopamine levels in the striatum of PD model of rats.	[304, 305]
HIF-1α	MPP^+^-intoxicated SH-SY5Y cells	Orexin-A is an inducer of HIF-1α that diminishes MPP^+^-induced cell injury.	[306, 307]
ATF4	Overexpressed or silenced ATF4 in cellular models of PD	Silencing ATF4 in neuronal PC12 cells boosts cell death in response to either 6-OHDA or MPP^+^. Overexpression of ATF4 decreases cell death caused by dopaminergic neuronal toxins.	[308, 309]
Hsp70	H4 cells transfected with α-syn	CBX treatment activates heat shock factor 1 and thereby induces Hsp70. Hsp70 abates α-syn aggregation and prevents α-syn-induced cytotoxicity.	[310, 311]
LAMP2A	SH-SY5Y neuroblastoma cell line stably expressing LAMP2A; primary cortical cultures with high CMA activity and a rat synucleinopathy model	Overexpressing LAMP2A enhances CMA activity and protects against neurotoxicity caused by α-syn. Co-injection of LAMP2A with α-syn reverses α-syn neurotoxicity	[312, 313]
TFEB	Induction of autophagy by CCI-779	CCI-779 inhibits mTOR, which increases nuclear translocation of TFEB, stimulates clearance of toxic oligomeric α-syn, and confers protection of nigral dopamine neurons against α-syn toxicity. Similarly, Rapamycin was observed to decrease Tau phosphorylation by inhibiting mTOR in senescence-accelerated OXYS rats.	[33, 314, 315]
PGC-1α	PGC-1α transgenic mice	Over-expressing PGC-1α in mice protects against MPTP-induced neuronal degeneration.	[316, 317]
T-type Ca^2+^ channels	6-OHDA lesioned rats	Local administration of a T-type Ca^2+^ channel antagonist significantly decreases locomotor deficits.	[318, 319]
HDAC6	PC-12 cells overexpressing human mutant (A53T) α-syn and SH-SY5Y cells intoxicated with MPP+	Overexpressing α-syn upregulates HDAC6 expression in close association with α-syn to form aggresome-like bodies. HDAC6 deficiency obstructs formation of aggresome-like bodies and restricts autophagy in response to MPP+-induced stress.	[320, 321]
PI3K/Akt signaling pathway	MPP^+^-intoxicated PC12 cells	Treatment with tetrahydroxystilbene glucoside attenuate loss of cell viability, release of lactate dehydrogenase (LDH), and inhibits apoptosis in a dose-dependent manner probably by activating the PI3K/Akt signaling pathway	[322, 323]

Abbreviations: BAG1: Bcl-2-associated athanogene-1, α-syn: α-synuclein, SN: substantia nigra, MPTP: 1-methyl-4-phenyl-1,2,3,6-tetrahydropyridine, CB2: cannabinoid type-2, LPS: lipopolysaccharide, 6-OHDA: 6-hydroxy dopamine, PD: Parkinson's disease, PDE7: phosphodiesterase-7, SNpc: substantia nigra pars compacta, SIRT2: Sirtuin-2, Trib3: Tribbles pseudokinase-3, MPP^+^: 1-methyl-4-phenylpyridinium, SK channel: small-conductance Ca^2+^-activated K^+^channel, VDR: vitamin D3 receptor, NMDAR: N-methyl-D-aspartate receptor, LHb: lateral habenula, HIF-1α: hypoxia inducible factor 1-alpha, ATF4: activating transcription factor 4, Hsp70: Heat shock protein-70, CBX: carbenoxolone, CMA: chaperone-mediated autophagic, TFEB: transcription factor EB, mTOR: mechanistic target of Rapamycin, PGC-1α: peroxisome proliferator-activated receptor-gamma coactivator-1α, HDAC6: histone deacetylase-6, PI3K/Akt: phosphatidylinositol 3-kinase/ protein kinase B

### Micro-RNA as a PD target

Micro-RNAs (miRNAs) are small non-coding, single stranded RNA molecules consisting of 22 nucleotides. miRNAs control gene expression by base pairing to mRNA and trigger translation repression [29]. Abnormal miRNA expression has been associated with various neurological disorders, such as AD, PD, Huntington's disease, amyotrophic lateral sclerosis, schizophrenia, and autism. Un-regulated miRNAs in patients suffering from PD could be used as biomarkers for early identification and monitoring disease progression. Ascertaining the role of miRNAs in cell processes and learning how disorganized miRNA expression accounts for neurological effects is crucial for discovering new therapeutic strategies for PD. miRNAs have great therapeutic potential, particularly if it can be demonstrated that a single miRNA can activate or inhibit several desired genes, making it possible to modify a whole disease phenotype by modifying a single miRNA molecule. Hence, understanding the mechanisms by which miRNAs participates in the pathogenesis of PD may offer novel targets to researchers to develop pioneering therapies [30]. Certain miRNAs are highly elevated in brain tissues of patients with PD. miRNA-301b, miRNA-373, miRNA-26b, miRNA-224, miRNA-21, and miRNA-106b are few of the miRNAs found in PD brain tissues that actively participate in the autophagy pathway carried out by chaperone [31]. Malfunctioning of this pathway has been suggested to disorder degradation of the alpha-synuclein (α-syn) protein, which contributes to Lewy Body (LB) pathology [32]. The effect of miRNA-128 overexpression, a negative regulator of transcription factor EB (TFEB), was examined by Decressac and Bjorklund. In this study, the AAV vector was used to overexpress miRNA-128 in midbrain dopaminergic neurons. They demonstrated that miRNA-128-facilitated suppression of TFEB, aggravated the toxicity of α-syn by inhibiting autophagy, and consequently favored formation of toxic oligomers [33].

Since, quantifying specific pathogenic proteins in the neuronal population is essential for survival of neurons involved in PD pathogenesis, evaluating the role of miRNAs is important for treating PD [34]. Choi et al. demonstrated that miRNA-7, confers protection in 1-methyl-4-phenylpyridinium (MPP^+^)-induced cytotoxicity to differentiated human neural progenitor ReNcell VM cells, primary mouse neurons and also to dopaminergic SH-SY5Y cells. With the help of quantitative proteomic analysis, Choi and colleagues also determined that RelA, which is a constituent of nuclear factor-kappaB (NF-κB), was downregulated by miRNA-7. Latter on RelA mRNA was confirmed as a target for miRNA-7 and is essential for MPP^+^-induced cell death. These outcomes describe a novel mechanism by which suppression of NF-κB is responsible for the cell death mechanism following MPP^+^-induced toxicity, and, thus, propose miRNA-7 as a therapeutic target for PD [35]. In a very recent study, kabaria and colleagues demonstrated that miRNA-7, decreases expression of Keap1 by targeting the 3′-untranslated region (UTR) of its mRNA in SH-SY5Y cells which consequently amplifies nuclear factor erythroid 2-related factor 2 (Nrf2) activity. In addition, miRNA-7 was found to augment the level of reduced form of glutathione and decrease the intracellular hydroperoxides level, suggesting its anti-oxidative effect. These conclusions signify to a novel mechanism by which miRNA-7 exhibits its cytoprotective effects by activating the Nrf2 pathway [36]. *In vitro* and *in vivo* data have confirmed that dopaminergic neurons depend profoundly on a functional miRNA network. In a study that examined the role of miRNAs in mammalian midbrain dopaminergic neurons, miRNA-133b was found to be precisely expressed in midbrain dopaminergic neurons of healthy individuals but midbrain tissue from patients with PD entirely lacked this type of miRNA. It was proposed that the development and operation of midbrain dopaminergic neurons is regulated by miRNA-133b through a negative-feedback mechanism, which contains paired-like homeodomain transcription factor Pitx3 [37].

Sequence analysis of human α-syn showed that the entire UTR of the α-syn gene is highly conserved, insinuating a role for miRNA regulation [38]. Until now, miRNA-153 and miRNA-7 are the only two miRNAs that have been shown to directly aim α-syn. These two miRNAs downregulate α-syn mRNA and protein levels by binding to the 3′-UTR of α-syn [39]. Additional cellular studies have demonstrated that miRNA-7 decreases α-syn-induced neurotoxicity in a cellular model [40]. Additionally, miRNA-29a, miRNA-1, and miRNA-22 are less expressed in patients with PD compared to control subjects [41]. Thus, indicating that specific miRNAs could serve as effective biomarkers in patients with PD. A similar study performed using plasma from patients with untreated PD and control subjects found seven upregulated miRNAs, such as miRNA-454, miRNA-125a-3p, miRNA-137, miRNA-181c, miRNA-193a-3p, miRNA-196b, and miRNA-331-5p in PD patients. Based on these evidences, miRNAs have tremendous potential to be developed as biomarkers or therapeutics for PD [42].

### Alpha7 nicotinic receptor as a PD target

Neuronal acetylcholine receptors (nAChR) are pentameric ligand-gated ion channels that consist of different combinations of α and β transmembrane subunits [43–45]. The α7 receptor is membrane-bound receptor and consists of five identical α subunits, with five agonist binding sites. α7 nAChRs are highly expressed in the hippocampus, medial habenula, thalamus, hypothalamus, geniculate nuclei, colliculi cortex, and amygdala, but are scarcely expressed in forebrain, striatum, medulla, and numerous brain nuclei [46]. The notion that nicotine might be useful to treat PD initially came from epidemiological data [47, 48]. The outcome of these experiments, combined with the discovery that nicotine enhances dopamine release [49, 50] suggested that nicotine might be responsible for the beneficial results of smoking in patients with PD. A protective role for α7 nAChRs against degeneration of dopaminergic neurons originated from experiments that used nicotine as a nAChR agonist [51–53]. Early reports by Janson et al. showed that nicotine dosed at or before the time of lesioning considerably increases nigral and striatal dopaminergic markers in rats [54, 55]. Later on, the neuroprotective ability of nicotine was established in several rodent models simulating damage to the dopaminergic nigrostriatal pathway. The evidence includes neuroprotection against 6-hydroxydopamine (6-OHDA)-induced nigrostriatal damage in rats [56] and protection against 1-methyl-4-phenyl-1,2,3,6-tetrahydropyridine (MPTP)-induced degeneration of nigrostriatal neurons in mice. Nevertheless, these results were not consistent enough to establish the neuroprotective potential of nicotine [57–59]. Subsequent experiments in parkinsonian nonhuman primates revealed that nicotine increased striatal dopaminergic components, including the DA transporter, tyrosine hydroxylase (TH), the vesicular monoamine transporter and DA [60, 61]. Data obtained from nonhuman primates with simulated PD strongly indicated the neuroprotective potential of nicotine. Other nAChRs subtypes, besides α7 nAChRs, are also protective against nigrostriatal damage. The α4β2 nAChR agonist ABT-089 confers neuroprotection in 6-OHDA-induced damage nigrostriatal neurons in rats [62]. In contrast, nicotine assisted neuroprotection against nigrostriatal damage was not reproduced in α4β2 nAChR knockout mice [63]. This result pinpoints the importance of β2 nAChRs for the effect of nicotine. One possible mechanism by which nicotine exhibits its protective role might be through chaperoning β2 nAChRs to the cell surface. Chaperoning could change the structures and functions of the endoplasmic reticulum, the secretory vesicles, and the Golgi apparatus of cells thereby reducing endoplasmic stress and enhancing cell survival [64, 65]. Drugs with different levels of agonistic efficacy, such as the allosteric α7 nAChR modulator galantamine and the α7 agonists ABT-107 and DMXB, are neuroprotective against 6-OHDA-induced damage to nigrostriatal neurons in rats [62, 66, 67]. Moreover, the α7 agonist PNU-282987 is also neuroprotective for MPTP-induced damage to nigrostriatal neurons in mice [68]. In contrast, the α7 nAChR antagonist methylycaconitine blocks the neuroprotective effect of nicotine [69]. These data indicate an important role for these receptors in protection against nigrostriatal damage. Nicotine may also be useful to manage levodopa-induced dyskinesias (LIDs). Experimental data from nonhuman primates and rodents indicate that drugs interacting with nAChRs may reduce LIDs [70]. Preliminary experiments with the general nAChR agonist nicotine showed a 60% decrease in LIDs in parkinsonian rodents and monkeys, indicating efficacy across species [52, 71–74]. Similarly varenicline, another general nAChR agonist [75], reduces LIDs by 50% [76]. Notably, pharmacological effects of nicotine were still observed in chronically treated nonhuman primates for over 1 year [73]. Nicotine decreases LIDs, regardless of route of administration. At least 1 month is required to observe the peak anti-dyskinetic effect of nicotine [74]. However, it also took 1 month to completely abolish the anti-dyskinetic effect of nicotine [76]. These observations suggest that long-term molecular changes are most likely responsible for mediating the reduction in LIDs by nicotine. For example, the α7-agonist ABT-107 decreases LIDs by 60%, which continued for several months [76]. Interestingly, the ABT-107-induced anti-dyskinetic effect persisted for about 1 month after termination, suggesting long-term molecular changes. Another α7 agonist ABT-126 produced similar results [77]. Dosing the α7 nAChR agonist AQW051 to Macaca fascicularis also yielded a 60% reduction in LIDs, with no further deterioration of parkinsonism [78], indicating the effectiveness of α7 nAChR agonists in nonhuman primates. In conclusion, drugs that are agonistic to α7 and β2 nAChR diminish LIDs by up to 60% with no harmful effects on parkinsonism. Since α7 nAChR might signify ideal drug target to improve LIDs in PD. Numerous intracellular mechanisms have been presented that facilitate the beneficial effects of activating α7 nAChRs against noxious insults. Mitogen-activated protein kinases have been associated with α7 nAChR assisted neuroprotection against a range of toxicities to PC12 cells, spinal cultures, and keratinocytes [79–82]. Further downstream mechanisms related with α7 nAChR assisted neuroprotection include increases heme oxygenase [83], phospholipase C [80], nerve growth factor [84], and proinflammatory cytokines such as interleukin-1β (IL-1β) and tumor necrosis factor-α (TNF-α) [85]. In contrast, nitric oxide (NO) [86], caspases, and reactive oxygen species (ROS) [83] are associated with the toxic effects of α7 nAChR. Taken together, these results suggest α7 nAChRs are important targets for developing therapeutic approaches for PD.

### Alpha synuclein as a PD target

Growing evidence from various experimental studies demonstrates that the α-syn protein, which is the main constituent of LBs, aggregate and accumulate intraneuronally in the brains of patients with PD. However, the association between this protein and the beginning of symptomatic behaviors linked with PD remains to be explored [87]. Reports suggest that expression of α-syn participates in dopamine biosynthesis by decreasing the action of TH or altering its phosphorylation [88, 89]. The functional ability of α-syn is very strictly related to its structure. Hence, understanding the structure and normal function of α-syn will help to learn about its involvement in PD. Human α-syn is a small acidic protein composed of 140 amino acids and is coded by the α-syn gene [90]. Recent data has revealed that α-syn at Ser129 is prominently phosphorylated in patients with PD [91]. Mutation in the α-syn gene, reduced rate of degradation or likely modifications of α-syn, such as missense mutations, truncations, or chemical modifications due to oxidative stress are some of the prime reasons behind misfolding and aggregation of α-syn. These mutations are focused at the α-syn N-terminus, indicating that they hinder regular cellular function [87]. Current *in vivo* outcome [92] have indicated that suppressing α-syn may protect dopaminergic neurons. Silencing the human α-syn gene with miRNA-30-hSNCA (a miRNA-30 transcript) at striatal dopaminergic terminals decreases the motor abnormalities seen in α-syn expressing rats and guards against damage to SN dopaminergic neurons. Conversely, efforts to silence the human α-syn gene with a small hairpin (sh)RNA in rat SN protected only against human α-syn-induced forelimb dysfunction but had no protective effect on DA neurons [93]. Moreover, high levels of shRNA α-syn are toxic to DA neurons, while lower levels of human α-syn gene silencing protect neighboring neurons. Taken together, these findings suggest that silencing the human α-syn gene may be a new therapeutic tool to control behavioral dysfunctions in PD. A number of clinical studies have shown the potential use of α-syn as a biomarker for PD in cerebrospinal fluid (CSF) [94].

Bearing in mind the several advantages of using biomarkers, researchers have begun to measure α-syn in peripheral organs of patients with PD. Wang, Gibbons et al. reported that patients with PD have increased levels of α-syn in cutaneous autonomic fibers [95]. This might clarify the incidence of autonomic dysfunction in patients with advanced PD [96]. Fresh data reveal that α-syn protein, its gene expression, and its signaling pathways is a challenging but effective way to alter motor behavioral insufficiencies and physiological deficits related with synucleinopathies. Additionally, many original compounds have been acknowledged as feasible to hinder or reverse the aggregation process [97]. Toth and coworkers used a combination of experimental and computational techniques [98] and discovered a small-molecule drug-like phenylsulfonamide compound (ELN484228) with the capability to bind monomeric α-syn and, therefore, reduce its transfer to mature synapses. These findings suggest that changing the properties of α-syn may have potential therapeutic benefits for battling PD. An alternative method to targeting this protein is to diminish Ser129 phosphorylation by obstructing the relevant kinases. Phosphorylation of α-syn appears to play an important role in the formation of the fibrillar aggregates causing PD pathology, since increased phosphorylation of α-syn is directly proportional to aggregation and toxic buildup in neurons [99]. Nevertheless, this tactic presents a potential restriction as numerous kinases are capable of phosphorylating α-syn. To tackle this issue, improvement of protein dephosphorylation has also been projected by Braithwaite and associates [100]. Hence, change in α-syn phosphorylation holds tremendous potential as a strategy to develop disease-modifying therapeutic interventions. Lately, numerous reports have described prion-like dispersal of misfolded α-syn [101–103]. Hence, there has been increased development of immunotherapies targeting clearance of α-syn aggregates and oligomers, [104, 105].

As toxic oligomeric forms of α-syn can enter and gather in the plasma membrane, get secreted and circulate extracellularly, it provides us a strong evidence for immunotherapy [104, 106] and also encourages development of oligomer specific antibodies [107]. Vaccination against α-syn can occur either by active or passive immunity. Active immunity involves stimulating the host to produce antibodies against α-syn aggregates, while passive immunization involves external administration of anti-α-syn antibodies to patients. The reason for the immunization to α-syn is to wash out these neurotoxic aggregates and impede neuron-to-neuron propagation [108]. In recent evidence, monoclonal antibody against α-syn was found to antagonize entry and cell-to-cell transfer of α-syn in primary neurons [109]. Further, Games and associates found that passively immunizing mThy1-α-syn transgenic mice with the truncated α-syn C-terminus mitigated axonal and synaptic pathology, salvaged loss of TH fibers in the striatum, and regulated motor and memory dysfunction [110]. Immunotherapy targeting α-syn remains puzzling, as fundamental mechanisms are not fully understood. Thus, further clinical work is needed. Several *in vivo* models of PD have revealed that neuroinflammation is not only an initial event but also fast-tracks the progression of nigral cell death. Yan and colleagues confirmed that dopaminergic neurodegeneration prompted by misfolded α-syn was intensified by activation of microglia through α-syn phagocytosis and release of cytokines and ROS [111]. α-syn released from injured dopaminergic neurons activates microglia and stimulates the release of proinflammatory mediators, causing the chronic and progressive dopaminergic neural degeneration linked with PD [112, 113]. Nevertheless, the relationship between PD-associated α-syn aggregation and microglial-induced neuroinflammation remains obscure. A recent article indicates that Toll-like receptor (TLR) 4 and TLR2 was observed to be essential for α-syn assisted activation of microglia [111, 114, 115]. Also, α-syn fibrils induced release of IL-1β from monocytes is facilitated by TLR2 [116]. Furthermore, the ability of microglia to detect misfolded α-syn increases the neurotoxic effect through the production of proinflammatory cytokines and ROS [117, 118]. Additionally, α-syn triggers constituents of innate and adaptive immune systems in patients with PD, which can alter pathological processes in animal models of PD [119, 120]. In fresh evidence by Thome and colleagues fractalkine signaling was observed to increase the inflammatory response in α-syn model of PD. Hence this report indicates that fractalkine is essential in the development of synucleinopathies, and could tentatively be a target for neuroprotective therapies for PD [121]. Therefore, studies are now focused on delineating the mechanisms and pathways linking neuroinflammation and α-syn.

### Rho kinase as a PD target

Rho is a small GTP-binding protein that plays a crucial role in many cellular functions. RhoA is a member of the Rho family and participates in cellular mechanisms that act on its direct downstream effector Rho-associated kinase (ROCK) [122]. Irregular activation of the RhoA/ROCK pathway is seen in models of inflammatory demyelinating diseases, stroke, spinal cord injury, AD, and other diseases [29, 123]. A number of key mechanisms associated with activation of ROCK also play a major role in the degeneration of dopaminergic neurons [122]. Inhibiting ROCK confers protection to dopaminergic neurons in a MPTP/MPP^+^-induced *in vitro* and *in vivo* model of PD [124–127]. ROCK inhibitors protect dopaminergic neurons in a primary neuroglia mesencephalic culture intoxicated with MPP^+^ [125–127]. Labandeira-Garcia and associates reported that NADPH-oxidase through NF-κB, triggers ROCK, which, in turn, stimulates NADPH-oxidase [122]. This finding is congruent with earlier experiments in peripheral cells showing that NADPH-oxidase-derived superoxide actuates NF-κB [128], and NF-κB stimulates ROCK [129]. More than a few experimental studies have shown that activating microglia and generating ROS with NADPH-oxidase represent the early phases of dopaminergic cell death and that both aspects act synergistically with other elements to induce dopaminergic cell death as the primary stage of PD pathology [130]. Additionally, ROCK and the angiotensin (AT) system help deciding the fate of dopaminergic neurons. AT1 and AT2 receptors and NADPH oxidase have been found in dopaminergic neurons and nigral glial population [131]. In line with these observations a decrease in AT1 receptor levels, decreased MPTP-induced expression of RhoA and ROCK activity in the mouse SN. In cultured cells, the additive effect of MPP^+^ and AT-II-induced dopaminergic neuron death was also antagonized by the ROCK inhibitor Y-27632, indicating a vital interaction between the AT-II/AT1 and the RhoA/ROCK II pathway in MPTP-induced dopaminergic neuron death [126]. This evidence reveals that activating ROCK and NADPH may be strongly involved in the AT-II-induced inflammatory response, which is blocked by ROCK inhibitors.

Labandeira-Garcia and coworkers demonstrated that estrogen impedes the MPTP or 6-OHDA-induced neuroinflammatory response and dopaminergic cell death [122]. Also, inhibiting the nigral rennin-AT system works in favor with the anti-inflammatory and neuroprotective effects of estrogen [132–134]. Reports by Rodriguez and Dominguez reconfirmed these findings by demonstrating the protective effect of the ROCK inhibitor Y-27632 in dopaminergic cell death induced by estrogen depletion [135]. In addition, candesartan an AT1 receptor antagonist, blocks the estrogen-induced increase in ROCK activity [135]. Taken together, these outcomes recommend that stimulating ROCK might play a crucial role in increased susceptibility of dopaminergic neurons after the reduction in estrogen. Latter it was found that this outcome was facilitated by stimulating the AT-II/AT1 pathway. Several mechanisms seem to be associated in ROCK-induced dopaminergic susceptibility and the neuroprotective effects induced by the ROCK inhibitors [122]. Many prospective ROCK targets in apoptotic signaling have been proposed, including interactions with the pro-apoptotic factors glycogen synthase kinase 3 beta, Bcl-2 proteins, and protein kinase B [136]. The axon-stabilizing effects in injured neurons may denote another mechanism of neuroprotection for dopaminergic neurons after inhibiting ROCK [137]. Concurrent treatment with a ROCK inhibitor substantially augmented the number of surviving dopaminergic neurons [125, 127]. Nevertheless, glial cells play an important role in the neuroprotective effects of ROCK inhibition on dopaminergic [124, 125, 127] and other neurons [138]. Inhibiting ROCK decreases cell size and number of filopodia associated with microglial activation. In addition, ROCK inhibitor subdues activation of NADPH-oxidase and blocks the release of inflammatory cytokines, such as TNF-α and IL-β [76, 127]. In conclusion, ROCK is associated with a large number of cellular processes; thus, several mechanisms may be responsible for its protective effect on dopaminergic neurons.

### Leucine-rich repeat kinase 2 as a PD target

Non-synonymous point mutations in the leucine-rich repeat kinase 2 (LRRK2/PARK8) gene is the leading genetic reason for autosomal dominant PD. Mechanisms specific to LRRK2 are being revealed with fresh discoveries of LRRK2 substrates [139]. The LRRK2 gene encodes a 286 kDa protein and is located on human chromosome 12. Cellular LRRK2 carries out numerous functions including modulation of protein translation [140, 141], changing microtubule dynamics [142], participating in endocytosis [143] and autophagy [144]. G2019S is the most common PD-associated LRKK2 mutation identified compared to other mutations. Majority of patients with LRRK2 PD lose SN neurons having fluctuating levels of LB inclusions, tau neurofibrillary tangles, or a combination of both [145]. Consequently, targeting LRRK2 is a striking option for developing PD therapeutics. Communication between the Roc-COR domains is significant for controlling LRRK2 GTPase activity because of the enzymatic capacity of LRRK2 mutants. Decreased GTPase activities in Y1699C and R1441C mutants suggest that the GTP-bound state is related with the disease. Hence, variations in LRRK2 GTPase activity either by obstructing the GTP-binding site of the ROC domain or by increasing GTPase activity embraces the therapeutic potential for LRRK2-associated PD. Nevertheless, no such study has attained this objective [146].

Mutations in the LRRK2 kinase domain change kinase activities, indicating that the pathogenicity of LRRK2 is facilitated through a non-kinase mechanism [146–148]. Therefore, the current attention on LRRK2 is based on inhibition of its kinase domain. Oxidative stress induced by LRRK2 was salvaged by DJ-1 in a neuroblastoma cell line [149], suggesting that antioxidants are potential inhibitors of LRRK2 kinase toxicity. The selection of small molecule inhibitors mostly depends on their capability to antagonize LRRK2 phosphorylation sites, such as, constitutive phospho sites, LRRK2 auto-phosphorylation sites, and those altered in the inhibitor-resistant mutant [150]. Numerous lead molecules have been identified for LRRK2 that are specific, potent, brain penetrating and have druggable attributes that will lead to a clinical trial [151]. Another promising possibility is to search for mutation-specific inhibitors that have no effect on wild-type LRRK2. However, the pathogenic role of mutant LRRK2 is not understood making the clinical endpoint difficult to predict [151]. Moreover, destabilizing microtubules has been proposed as a junctional point of idiopathic and genetic forms of Parkinsonism. As microtubule dysfunction occurs in patients with PD and mitigating this flaw restores control of the PD phenotype [142], controlling microtubule dynamics could serve as a potential therapeutic target. LRRK2 is also strongly associated with autophagy [152] and endocytosis [153]. Therefore, the endolysosomal and autophagosome-lysosome pathways are prospective target points to correct the effects of LRRK2. Use of LRRK2 inhibitors as a therapeutic option needs to be investigated in-depth, as LRRK2 significantly participates in immune function, metabolism, and kidney homeostasis [154].

### Nuclear receptor related 1 protein as a PD target

Nuclear receptor related 1 protein (Nurr1) is a member of the ligand-stimulated transcription factors called nuclear receptors. Nurr1 does not have a hydrophobic site for ligand binding as in other nuclear receptors; thus, nuclear receptor function is ligand-independent [155, 156]. Nurr1 plays an important role in the development and specification of midbrain DA neurons throughout life [157]. Deficiency of Nurr1 in developed DA neurons results a decrease in DA neuron markers and motor impairments simulating early symptoms of PD. Removing Nurr1 from grown-up rodents results [158] in decreased expression of genes related with oxidative phosphorylation and mitochondrial function, suggesting a role for Nurr1 in the preservation of midbrain dopaminergic neurons [159–161]. Puigserver and colleagues performed a large meta-analysis of genome-wide gene expression studies and revealed that genes encrypting proteins participating in oxidative phosphorylation are highly dysfunctional in the remaining dopaminergic neurons of patients with PD [162]. The precise mechanism by which Nurr1 activates or inhibits these mitochondrial genes is not clearly known. However, other transcription factors essential for expressing nuclear respiratory genes, such as the nuclear respiratory factors NrF1 and NrF2, or the transcriptional co-activator peroxisome proliferator-activated receptor-γ co-activator 1α, which is the chief controller of mitochondrial biogenesis and cellular respiration, might functionally bind with Nurr1 [162]. Neuroprotective effect by CREB in neurons exposed to oxidative stress and the regulation of neuroprotective genes is facilitated by Nurr1 [163]. Also, increased expression of survival-promoting brain-derived neurotrophic factor is facilitated by increased Nurr1 expression induced by the NMDA receptor [164]. Nurr1 agonist increases transcriptional activation of dopaminergic specific genes and decreases the expression of proinflammatory genes in microglia [165]. Nurr1 is identified to be activated in mouse microglia *in vivo* after stereotaxic injection of lipopolysaccharide (LPS). Apart from having vital roles within dopaminergic neurons, Nurr1 has been projected to be part of an anti-inflammatory pathway in astrocytes and microglia that guard dopaminergic neurons from inflammation induced cytotoxicity [166]. In line with these reports, treatment with a Nurr1 agonist conferred neuroprotective and anti-inflammatory effects in 6-OHDA lesion model of PD [167]. Lately, pathologic α-syn aggregates were found to downregulate Nurr and its transcriptional targets in midbrain DA neurons [119, 168]. Numerous additional genes may be downregulated due to the toxicity of α-syn. α-Syn may impede glial cell-derived neurotrophic factor (GDNF)-signaling by inhibiting Nurr1 and its transcriptional target Ret [169]. Overexpressing α-syn blocks GDNF signaling and inhibits the ability of GDNF to protect dopaminergic neurons against α-syn-induced toxicity [170]. Taken together, these findings suggest that the GDNF-Ret-Nurr1 pathway is an exciting target for PD therapeutic interventions. Outcomes from various experiments have suggested that nuclear α-syn may be responsible, at least in part, for the pathological process, and that Nurr1 is one of its targets that is affected directly or indirectly [171, 172]. Therefore, decrease in expression of Nurr1 could spur dysfunction of dopaminergic neurons and collude the progression of PD; thereby making this protein a promising therapeutic target for PD [169].

### Glucagon-like peptide 1 receptor as a PD target

Currently there has been a gush of interest in glucagon-like peptide 1 receptor (GLP-1R) as a prospective target for PD therapies. Under normal physiological conditions, the GLP-1 is released from intestinal epithelial cells in anticipation of high glucose and acts on pancreatic β-cells, liver, and muscle to reduce glucose levels. Several GLP-1R agonists have been used to treat type-II diabetes. Various experiments have indicated a connection between PD and type-II diabetes, as both diseases share molecular networks, such as inflammation [173]. GLP-1R agonist exenatide has growth factor-like properties and numerous positive effects in animal models of neurodegenerative disease and acute brain injury, such as preserving synapse plasticity, stimulating neurogenesis, decreasing protein aggregation and inflammation [174]. Although the fundamental mechanisms are not fully clarified, one effect of GLP-1R stimulation is to trigger the transcription factor cyclic adenosine monophosphate response element binding protein, which is associated with neuronal survival and synaptic plasticity. A recent clinical trial involving patients with PD administered daily injections of exenatide for 12 months. Exenatide was found to improve motor and cognitive functions [175] over 12 months even though the drug was eliminated from the system [176]. Although these data hints at a disease-modifying effect, the results should be considered cautiously, as control patients were not given placebo. Presently, a controlled phase II trial in patients with PD is ongoing, using a slow-release form of exenatide (Bydureon), which is taken in a once/week injection. Even if exenatide has shown encouraging results, several other pharmacological approaches targeting GLP-1R are marketed for type-II diabetes and possibly have a more promising profile. For example, the GLP-1R agonists liraglutide and lixisenatide have lengthy half-lives, are effective in *in vivo* AD models [177–179], and can normalize Ca^2+^ levels in human neuroblastoma cells [180]. In the immediate future, several trials will aim to achieve disease modification in PD through chronic dosing of different GLP-1R agonist.

### Acid-sensing ion channel as a PD target

Conserving the physiological pH of interstitial fluid is critical for normal cellular functions. Tissue acidosis is a common pathological variation causing abnormal activation of acid-sensing ion channels (ASICs), which may considerably contribute to mitochondrial dysfunction, inflammation, and other pathological mechanisms, including stroke, pain, and psychiatric conditions. Hence, it has become clear that ASICs are important in the development of neurological diseases [181]. Intra and extracellular pH is sustained between 7.3-7.0 under normal physiological conditions. However, increased neuronal excitability changes cellular pH to trigger various ion channels and receptors in cell membranes, including voltage-gated and ligand-gated ion channels [182, 183]. ASICs, which were first cloned by Waldmann and associates [184], are stimulated in response to acidic extracellular pH [185]. Nevertheless, the practical roles of ASICs in central and peripheral components of the nervous system remain to be determined. At present, neurodegenerative diseases including PD and AD, are treatable but can't be cured by existing mode of treatment. Pre-clinical and clinical studies have suggested that overload of Ca^2+^, oxidative stress, mitochondrial dysfunction, energy metabolism, and acidosis are involved in neurodegenerative processes. These mechanisms frequently result in tissue acidification causing lactic acidosis, which further exacerbates neuronal damage. Similar observations have also been reported in the brains of patients with PD and in the typical MPTP-induced PD animal model [186, 187].

One study showed that mitochondrial ASIC1a might be a significant modulator of mitochondrial permeability transition pores and increase neuronal death due to oxidative stress [188]. Furthermore, Arias et al. discovered that MPTP-treated mice have brain acidosis and that treatment with the ASIC inhibitors amiloride and PcTx-1 confers protection against degeneration of SN neurons by preventing apoptosis and by decreasing DA and its transporter [189]. Moreover, the absence of endogenous parkin protein or mutations in the parkin gene cause irregular ASIC currents resulting in protein degradation and dopaminergic neuronal injury, indicating that ASIC currents may facilitate the essential pathology in PD [190]. Exposing rat microglial cells to LPS also increases ASIC1 and ASIC2a expression levels and stimulates production of inflammatory cytokines [170]. These reports indicate that controlling microglial ASIC function might control the disease pathology in PD.

### Dopamine heteroreceptor complexes as PD target

The finding that DA-1 receptor (D1R) and D2R exists in the brain in the form of heteroreceptor complexes helped in understanding the therapeutic actions and side effects of L-DOPA and DA receptor agonists in the treatment of PD [191, 192]. Various heteroreceptors, including adenosine-A2A-D2R (A2AR-D2R), A2AR-D2R-metabotrophic glutamate receptor (A2AR-D2R-mGluR5), D2R-N-methyl-D-aspartate receptor (D2R-NMDAR), D1R-D3R, A1R-D1R, D1R-NMDAR, and A1R-D1R-D3R heteroreceptor complexes could be potential targets for alleviating the side effect effects associated with L-DOPA. The finding that coactivation of the D1R and D2R protomers leads to calcium signaling in the striatum [193] suggested that the mental side effects of DA agonist treatment, such as gambling, psychosis, and hallucinations, can include stimulation of the D1R and D2R protomers of this heteroreceptor complex.

#### D1R-D3R heteroreceptor complexes

An abnormal increase in D1R protomer signaling produces LID [194]. Therefore, antagonizing over-activated D1R protomer signaling in various types of homo and heteroreceptor complexes could be a promising target for treating LID.

#### D2R-D3R heteroreceptor complexes

A detailed understanding of the function and potential dysfunction D2R-D4.2R and D2R-D4.4R heteroreceptor complexes in PD is lacking. However, due to the activity of antiparkinsonian drugs, such as L-DOPA and apomorphine, on D4Rs, they also act on D2R-D4.2R and D2R-D4.4R heteroreceptor complexes, which could open new doors by increasing the plasticity of responses to L-DOPA treatment [195].

#### A2AR-D2R heteroreceptor complexes

Fuxe and colleagues demonstrated antagonistic A2AR-D2R interactions at the level of D2R recognition in striatum from naive and hemiparkinson rats [196] and also at the level of the striatopallidal GABA pathway and its brain circuits in a naive rat model of PD [197]. These findings lead to the hypothesis that A2AR antagonists could be novel antiparkinsonian drugs by acting the A2AR homomer and protomer in the striatopallidal GABA neurons through antagonistic A2AR-D2R receptor-receptor interactions [198] resulting in antidyskinetic actions, which could help lessen the wearing off of the therapeutic effects of L-DOPA.

#### A2AR-D2R-mGluR5 heteroreceptor complexes

Due to the supremacy of co-activated A2AR and GluR5R receptor protomer signaling, chronic treatment with D2R agonists and L-DOPA is been often observed to produce a strong inhibition of D2R protomer signaling in A2AR-D2R-mGluR5 heteroreceptor complexes [199]. Therefore in the treatment of PD, we might need to aim A2AR and mGluR5 receptors located on striato-pallidal GABA neurons by co-antagonizing them via using heterobivalent compounds. These compounds will encompass A2A antagonistic and mGlu5 antagonist/negative allosteric modulator properties to eliminate the inhibition on D2R protomer signaling [195].

#### D2R-NMDAR (NR2B containing) heteroreceptor complexes

Liu and coworkers established that the NR2B subunit of the NMDAR directly binds with the D2R [200]. Based on this finding, it is plausible to consider aiming D2R-NMDAR (NR2B containing) heteroreceptor complexes by co-treatment of a NR2B-selective NMDA antagonist with an mGluR5 antagonist/negative allosteric mGluR5 modulator to treat PD.

#### A1R-D1R heteroreceptor complexes

Similar to antagonistic A2AR-D2R interactions, there is also an existence for antagonistic interactions between A1R-D1R in the modulation of GABA release in hemi-parkinsonian rat [198] and also in D1R agonist-stimulated motor effects in rodents [201]. Interestingly, it is worth to note that A1R agonists in rabbits can neutralize D1R agonist-stimulated oral dyskinesias [202]. Therefore with respect to the function of D1Rs in facilitating LIDs [203], these findings suggest the possibility that initial co-treatment with A1R agonists in A1RD1R heteroreceptor complexes can neutralize the development of LIDs. Hence, pharmacology of A1R should be investigated in cellular models with A1R-D1R and A1R-D1R-D3R heteroreceptor complexes as targets. Cumulatively the existing investigations on D1R and D2R heteroreceptor complexes in the basal ganglia propose a novel molecular mechanism for understanding the loss of efficacy of L-DOPA and DA receptor agonists and the development of dyskinesias by L-DOPA and DA receptor agonist.

In addition to the above mentioned heterereceptor complexes, growth hormone secretagogue receptor (GHSR1a) also is documented to form dimer with D1R. GHSR1a is a biological target of ghrelin peptide that is extensively disseminated throughout the brain. GHSR1a and D1R have been demonstrated to be co-expressed in areas including ventral tegmental areas, SN, and midbrain. Dimerization of D1R with GHSR1a amplifies cAMP signaling and subsequently D1R signaling. This suggests that dimerization is associated with mood, learning, and memory [204].

### ATP13A2 as a PD target

The significance of ATP13A2 also known as PARK9 in PD has increased with the finding that mutations in this gene cause Kufor-Rakeb syndrome (KRS) which is an autosomal recessive, juvenile-onset form of parkinsonism [205]. With the knowledge that ATP13A2 is a disease-insinuating gene; a series of laboratory studies were initiated to unravel the molecular function and classify the pathophysiological mechanisms that result in the clinical phenotype. Early indications from various disease models suggested that ATP13A2 participates in Mn^+2^ and Zn^+2^ metabolism [206, 207], mitochondrial bioenergetics [208–210], and in the autophagy-lysosomal pathway [209, 211, 212]. Moreover, ATP13A2 has been exposed to control α-syn metabolism, one of the major components of LB [212]. Detecting this monogenic form of PD has spurred to a number of experiments examining the role of this gene in sporadic PD. Numerous solo heterozygous ATP13A2 mutations have been recognized with greater incidence in early-onset PD as compared to that in healthy controls, indicating that these mutations are an age-of-onset modifier or a risk factor for PD [213, 214]. Preliminary indications signifying the role of ATP13A2 in sporadic PD came from the observation that ATP13A2 mRNA levels increase in surviving dopaminergic SN neurons from brains of patients with sporadic PD [215]. Another distinct finding also confirmed a noticeable increase in the ATP13A2 protein in the SN and other brain regions of patients with sporadic PD [210]. Consequent studies consistently reported substantial changes in ATP13A2 levels in the brains of patients with sporadic PD [211, 216]. Additional evidence for a role of ATP13A2 in sporadic PD comes from the consequences of its absence. Functional ATP13A2 appears to be indispensable for breaking down hoarded or aggregated α-syn [212, 217]. Any slight deviation in its competence over a lifetime could contribute to the progression of synucleinopathy. Moreover, participation of ATP13A2 in externalization of α-syn through exosomes may be important in the development of PD, either by removing α-syn from the cytoplasm or by disseminating the protein into neighboring cells [207]. ATP13A2 may also unravel the relationship between α-syn metabolism and mitochondrial dysfunction in patients with sporadic PD. Mounting evidence puts mitochondrial dysfunction during neurodegeneration at the center of both familial [218, 219] and sporadic PD [220, 221]. Cell lines derived from a patient with KRS [208, 222] and mammalian ATP13A2-silenced cell lines [209, 210] had pathogenic changes in mitochondria function. The function of ATP13A2 in lysosomes and mitochondria appears to be directly connected to its role as a Zn^+2^ transporter and signifies the common fiber that connects these two apparently dissimilar organelle systems in PD [217, 222]. Dysregulation of zinc connected to insufficiency of ATP13A2 could clarify the established link between sporadic PD, higher brain zinc levels, and other tissues [223, 224]. Taken together these evidences back the participation of ATP13A2 in numerous overlapping pathogenic pathways intimately associated with PD. Additional examination on the expression levels of ATP13A2 as a target and their functional significances in these mechanisms, using suitable PD models, will be essential to widen the learning of ATP13A2 in PD pathogenesis.

### Glutaredoxin as a PD target

Mutations in LRRK2 are related to autosomal dominant PD, and many of these mutations are observed to increase cellular ROS levels. Therefore, antioxidant proteins are essential to reinstate the redox balance and preserve cell viability. Studies in the past decade have begun to establish the prominence of redox proteins in facilitating neuroprotection in PD models [225]. Glutaredoxin (Grx) is small redox enzyme that precisely catalyzes the removal of glutathione from cysteine residues [226], thereby reinstating the function of proteins whose function is altered upon glutathionylation [227]. Grx1 is a well-recognized major deglutathionylating enzyme, exhibiting about 5000-fold greater catalytic competence for deglutathionylation compared to Trx1 [228]. The primary finding demonstarting that Grx1 participates in protecting dopaminergic neurons was observed when Grx1 mRNA and protein levels were elevated in mouse brain homogenate post-MPTP treatment [229], indicating a homeostatic upregulation of the chemical intoxication. Another study reported that female mice have higher Grx1 content than that of males and are more resistant to MPTP-induced dopaminergic cell death [230]. Supplementary experiments provided additional evidence that Grx1 facilitates neuronal protection. Treating SH-SY5Y cells with the pro-oxidant drug L-DOPA increased apoptosis. After examining the mechanism of drug-induced cell death, Grx1 was particularly inactivated compared to other redox enzymes [231]. This finding led to the suggestion that Grx1 plays an important role upholding neuronal cell viability. To check this concept, Grx1 was silenced in SH-SY5Y cells. Cells exposed to Grx1-siRNA presented an increased level of apoptosis, similar to control cells with non-targeting siRNA [231]. This result was also confirmed by Grx1 knockdown of Neuro-2a cells via shRNA, resulting in cell death [232]. Taken together, these results suggest that Grx1 plays a neuroprotective role in cultured cells. Although promising results have been seen *in vitro*, the neuroprotective role of Grx1 has not been investigated *in vivo* and its implications for PD in humans [233]. Johnson and colleagues examined the role of Grx1 in conferring protection to dopaminergic neurons in a C. elegans PD model. C. elegans worms overexpressing α-syn, TH, LRRK2-G2019S, and LRRK2-R1441C in dopaminergic neurons, simulating both familial and sporadic PD model, were crisscrossed with worms missing the Grx1 homolog called GLRX-10. Each of the hybrid worm lines missing the Grx1 homolog displayed a significantly more severe PD phenotype compared to the control worms with endogenous GLRX-10. Moreover, re-expression of wild-type GLRX-10, in the dopaminergic neurons of the GLRX-10^−/−^/LRRK2-R1441C worms salvaged the exacerbated PD-like phenotypes [225]. Johnson and coworkers also inspected Grx1 content in brain samples of patients with and without PD. Immunoblot analysis of midbrain homogenates showed an overall reduction in the Grx1 protein in the midbrains of patients with PD compared to control subjects. Additionally, midbrain tissue slices revealed that more dopaminergic neurons were devoid of Grx1 in patients with PD compared to those in control subjects. Overall, these data contributes to the *in vivo* evidence that Grx1 protects dopaminergic neurons in familial and sporadic PD. Also, it reveals that Grx1 protein content is reduced in PD brains, indicating that decreased Grx1 with aging precedes PD [225]. Furthermore, Johnson and colleagues found that loss of the Grx1 homolog in worms aggravated LRRK2-induced dopaminergic neuronal toxicity. Over production of DA or α-syn was observed to be responsible for the loss of Grx1 which further worsened PD phenotypes in models of sporadic PD. However, re-expression of the catalytically active site of the Grx1 homolog avoided the intensified toxicity. Largely these data noticeably implicates that reversible protein glutathionylation is the most likely mechanistic base for the catalytic role of Grx1 in facilitating protection to dopaminergic neuronal against the oxidative stress associated with overexpression of mutant α-syn or LRRK2. Thus, elimination of the glutathione modification from vital regulatory proteins by Grx1 is obligatory to reinstate their function and preserve cellular homeostasis and cell survival. Based on the available data and the need to combat oxidative stress in PD, Grx1 can serve as a novel therapeutic strategy for PD [225].

### Metabotropic glutamate receptor as a PD target

Nickols and Conn have reported all pertinent preclinical indications that will lead to expanding mGlu4 receptor-positive allosteric modulators (PAMs) and mGlu5 receptor negative allosteric modulators (NAMs) as potential anti-parkinsonian drugs [234]. One major drawback in the existing treatment of PD is motor fluctuations and LIDs. No treatments are available for LIDs except weak NMDA channel blockers and amantadine. In a recent report by Xia and associates, it was demonstrated that the selective mGluR5 antagonist, 2-methyl-6- (phenylethynyl) pyridine in the presence of rotenone intoxication, mitigates DNA damage in MN9D dopaminergic neurons by decreasing intracellular calcium release which further decreases mitochondrial dysfunction and endoplasmic reticulum stress caused by ROS [235]. mGlu5 receptor NAMs have demonstrated *in vivo* activity by decreasing both LIDs and parkinsonian motor symptoms. These drugs may protect nigral dopaminergic neurons in rodents and non-human primates [236, 237]. Mavoglurant (AFQ056) is an mGlu5 receptor NAM that exhibits substantial anti-dyskinetic activity without restricting the therapeutic efficacy of L-DOPA in preliminary, Phase 2a, and Phase 2b clinical trials [238]. However, mavoglurant was discontinued, as it was not found active in further studies. Due to the phenotypic response by glutamate in manifesting motor behavior, it is plausible that mGlu5 receptor blockade is a reliable strategy to treat LIDs. Similarly, dipraglurant, another mGlu5 receptor NAM, showed good safety and acceptability profile with noteworthy efficacy against LIDs in a Phase 2a study [238]. Nevertheless, all of these studies were short-term and it remains unclear whether mGlu5 receptor NAMs can change synaptic plasticity underlying LIDs or whether they have symptomatic effects.

The mGlu4 receptor is another recognized target for treating PD, and several orthosteric agonists and mGlu4 receptor PAMs have presented symptomatic efficacy in experimental models of parkinsonism [234, 239]. Stimulating mGlu4 receptors could attenuate the degeneration of dopaminergic neurons in PD [240, 241]. Jeff Conn and colleagues discovered that mGlu4 receptors form homodimers at striatopallidal synapses, whereas they form inter-group heterodimers with mGlu2 receptors at corticostriatal synapses. PHCCC, an mGlu4 receptor PAM, selectively triggers mGlu4 receptor homodimers, whereas other PAMs, such as VU0155041 and Lu AF21934, also stimulate mGlu4/mGlu2 heteromers [242]. It will be exciting to associate the activity of the two dissimilar categories of PAMs in models of parkinsonism. mGlu3 receptor PAMs still have the potential as disease-modifying agents in PD since, mGlu3 receptors activate the production of neurotrophic factors such as GDNF and transforming growth factor-β [243].

### Niacin receptor 1 as a PD target

Niacin receptor 1 (GPR109A) is a high-affinity niacin receptor that is overexpressed in the SN of patients with PD. Niacin is the primary source to synthesize NAD-NADH, which is required for DA production. Hence, niacin supplementation might assist three purposes. First, DA synthesis in the striatum could increase by supplying NADPH, and a higher NAD/NADH ratio would boost mitochondrial functions and decrease inflammation through GPR109A-related mechanisms [244]. The neuroprotective role of niacin in PD was suggested by an unreliable report [245]. Furthermore, various reports have documented that chronic use of levodopa decreases the level of niacin by interfering with tryptophan breakdown. Tryptophan metabolism itself is impaired in patients with untreated PD [246, 247]. Long-term niacin deficiency causes mitochondrial dysfunction, oxidative stress, and death of DA producing cells in the SN [248]. Therefore, the decrease in mitochondrial function may actually be an outcome of inflammation resulting from chronic niacin deficiency. Niacin is a very important factor for producing NADPH. An altered NAD-NADH ratio is implicated in ATP depletion and mitochondrial dysfunction, resulting in neuronal death in neurodegenerative diseases, such as PD [244]. NADPH is required to synthesize of tetrahydrofolate, which is crucial coenzyme required for DA synthesis. With decreased levels of niacin in PD, NADPH levels decrease and lessen striatal DA production. Therefore, it is reasonable to deliver a natural source for the coenzyme rather than supplying a ready-made coenzyme, which is niacin [244]. Wakade and colleagues suggested that niacin plays a significant role protecting dopaminergic neurons in PD via the GPR109A pathway, either by augmenting blood supply to hypoperfused areas in the brain or by increasing anti-inflammatory mechanisms. GPR109A is also called hydroxycarboxylic acid receptor 2 (HCAR2), HM74a in humans, and PUMA-G in mice. GPR109A is a G-protein-coupled high-affinity niacin receptor [249] and beta-hydroxyl butyrate is its physiological ligand [250]. GPR109A is expressed in different human tissues, including the brain [251]. However, immune cells express the highest amount of the GPR109A protein in humans [251]. GPR109A and its ligands have been recognized for their anti-inflammatory properties in a range of *in vivo* and *in vitro* experimental models, including the retina, skin, and gut. Although the anti-inflammatory effect of GPR109A has not been shown in the brain, one report has indicated that GPR109A agonists subdue LPS-induced inflammation via the NF-κB pathway in the gut [252]. Also, activation of GPR109A in mouse macrophages by interferon gamma substantiates the role of GPR109A in inflammation [253]. However knocking down GPR109A has no effect on NAD-NADH or DA production. NADH is required for DA synthesis and cultured PC12 cells show increased TH and DA levels [254] by augmenting the reuse of quinonoid dihydrobiopterin to tetrahydrobiopterin [255]. Additionally, systemic administration of NADH increases urinary excretion of homovanillinic acid, which is suggestive of increased endogenous L-DOPA synthesis [256]. Therefore, DA may be augmented directly by supplying NADH *in vitro*. Niacin is involved in many other signaling pathways, including increasing nitric oxide, arachidonic acid, and prostaglandin D2 and E2 levels [257, 258]. Niacin also stimulates adiponectin levels. Adiponectin may be neuroprotective by reducing oxidative damage [259, 260]. Therefore, GPR109A could be useful to battle acute or chronic inflammation in PD [244] and reduce PD progression.

### Apolipoprotein E as a PD target

The Apolipoprotein E (APOE) gene is situated on chromosome 19 and encrypts a plasma glycoprotein composed of 299 amino acids, which is linked with low density lipoprotein (LDL), very low density lipoprotein (VLDL), and high-density lipoprotein (HDL) [261]. APOE is synthesized ubiquitously in the body, including the brain, liver, skin, and in macrophages [262]. Several major APOE isoforms have been described, such as E2, E3, and E4. Six different APOE phenotypes are detectable due to two single nucleotide polymorphisms at amino acid positions 112 and 158. These mutations could change the protein charge and stability; thus, inducing unique physiological functions. APOE decreases blood lipid and lipoprotein levels. APOE acts as a ligand for members of LDL receptor family and mediates removal of lipoproteins from the circulation for excretion via the liver. APOE forms VLDL and chylomicrons and changes the activity of other lipid metabolism-influenced proteins and enzymes, such as lipoprotein and lipase hepatic lipase. New data suggest that APOE and its isoform function beyond lipid metabolism to encompass maintenance of normal brain function [263]. Numerous APOE isoforms with structural differences have been discovered that serve functions in mitochondrial signaling, neuronal signaling, neuroinflammation, brain lipid transport, and glucose metabolism. Understanding the mutations in APOE, their isoforms, and their structural properties will help determine role in various diseases and to advance therapeutic strategies. Targeting APOE could help with risk assessment, diagnosis, prevention, and treatment of various neurodegenerative and cardiovascular diseases in humans. APOE is mostly manufactured by astrocytes or by neurons under certain pathological conditions [264, 265]. The human brain comprises up to 25% of the body's cholesterol, which is indispensable for myelin production, function, and its integrity. Cholesterol is a crucial constituent of axonal growth and synaptic formation, which are vital for learning and memory [266, 267]. Regulating cholesterol in the central nervous system (CNS) is independent of the peripheral system. Any dysfunction in CNS cholesterol could have influence on aging and the development of certain neurodegenerative diseases. APOE delivers cholesterol to neurons [268, 269], but the BBB limits the interchange of lipoproteins and APOE between the peripheral and CNS systems. One study reported that brain injury causes an increase in APOE protein content in the brain [270] but the mechanisms relating APOE to all of these biological processes have been elusive. Recently, association between APOE and PD has been demonstrated [271, 272]. Maximum studies were unsuccessful to testify any relation between APOE ε4 and vulnerability to PD and PD-associated cognitive dysfunction [273, 274]. Various experiments have shown that APOE ε4 is a risk factor for age of onset and reduced cognitive functioning associated with PD. Though, ε2 is considered a weak or inconsistent risk factor for PD [274–277], but a meta-analysis suggested that the ε2 allele is related with advanced risk for PD [72, 278]. However, another study reported that the ε4 allele is associated with PD development [271]. Until now, experiments concentrating on the role of APOE in PD remain largely inconclusive and more studies are needed to determine whether APOE is a PD target.

## CONCLUSION AND PERSPECTIVE

**Figure 1:**
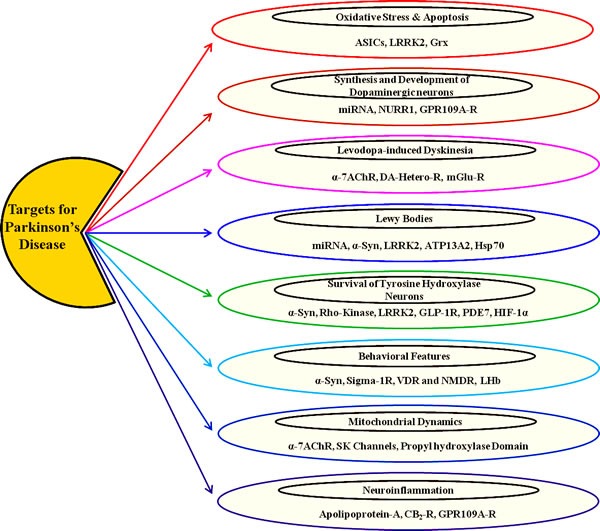
Preclinical Targets for Parkinson's disease (PD) As illustrated, there are eight capsules, each signifying various targets acting on particular pathological process and/or outcome of PD.

Irrespective of the intensive research from the past few decades, all existing therapies for PD are focused on providing symptomatic relief and not towards achieving neuroprotective or disease-modifying strategies. Therefore, there is a serious need for an exemplar shift in discovering novel and effective targets that cures or stops the advancement of PD. However, discovering a definitive cure for PD is a highly difficult task, as many obstacles need to be handled for the development of effective disease-modifying or neuroprotective treatment. Novel targets can be discovered by the development of translationally accurate disease models, as current models of PD do not sufficiently simulate the advanced nature of the disease. Furthermore, a multi-target therapeutic approach (polypharmacology) might also combat the disease from various pathways, thus significantly improving the likelihood of disease modification. In our existing write-up and also as illustrated in Figure 1, we have summarized recent findings concerning preclinical investigations of various targets linked to PD. Current developments in understanding the molecular and cellular mechanisms of PD have facilitated us with a quick progress in identifying new PD targets. Additionally, multiple models can also be used to measure whether a target is capable of impacting different stages involved in PD pathogenesis. Quite a few targets have been examined in clinical trials designed to evaluate disease modification in PD, but all haven't been satisfactorily fruitful. Over the past 3 years, clinical trials exploring the possibility of coenzyme Q10, creatine, dopaminergic receptor, adeno-associated virus serotype 2 (AAV)-neuturin, and PPAR-γ receptors have reported negative outcomes. Regardless of these drawbacks, clinical trials presently are testing the potential of targets such as, calcium channel blocker, adenosine receptor, neuronal nicotinic receptor, glutathione, AAV2-GDNF, as well as active and passive immunization against α-syn as disease-modifying therapies. We believe that rather than achieving short term benefits for PD, setting long-terms goals by carrying out integrative research by merging or collaborating academic and industrial outcomes would manifest an actual therapy for PD.
